# Raised seasonal temperatures reinforce autumn *Varroa destructor* infestation in honey bee colonies

**DOI:** 10.1038/s41598-021-01369-1

**Published:** 2021-11-15

**Authors:** Szymon Smoliński, Aleksandra Langowska, Adam Glazaczow

**Affiliations:** 1 Department of Fisheries Resources, National Marine Fisheries Research Institute, Kołłątaja 1, 81-332 Gdynia, Poland; 2 Department of Zoology, Section of Apidology, Poznań University of Life Sciences, Wojska Polskiego 71c, 60-628 Poznan, Poland; 3 Department of Systematic Zoology, Adam Mickiewicz University, Uniwersytetu Poznańskiego 6, 61-614 Poznan, Poland

**Keywords:** Climate-change ecology, Ecological modelling

## Abstract

*Varroa destructor* is the main pest of the honey bee *Apis mellifera*, causing colony losses. We investigated the effect of temperature on the autumn abundance of *V. destructor* in bee colonies over 1991–2020 in Central Europe. We tested the hypothesis that temperature can affect autumn mite populations with different time-lags modulating the bee abundance and brood availability. We showed that raised spring (March–May) and autumn (October) temperatures reinforce autumn *V. destructor* infestation in the bee colonies. The critical temperature signals embrace periods of bee activity, i.e., just after the first cleansing flights and just before the last observed bee flights, but no direct effects of phenological changes on *V. destructor* abundance were found. These effects were potentially associated with increased bee reproduction in the specific periods of the year and not with the extended period of activity or accelerated spring onset. We found significant effects of autumn bee abundance, autumn capped brood abundance, and the number of colonies merged on autumn mite infestation. We also observed differences in *V. destructor* abundance between bees derived from different subspecies. We indicated that climatic effects, through influence on the bee abundance and brood availability, are one of the main drivers regulating *V. destructor* abundance.

## Introduction

Temperature alterations caused by human influence over the last century are among the most significant elements of recent global climate change^1^. A growing body of literature shows that these changes have affected a variety of organisms in different locations worldwide^2^, including, e.g., phenological alterations or shifts in species distribution ranges^3,4^. Observed temperature changes influence organisms not only in a direct way but also indirectly by the alteration of ecological interactions between different taxa at the same or adjacent trophic levels^5,6^. Therefore, the projected further increase in the global temperature, can impact the structure and functioning of whole communities or ecosystems^1,2^. Improving our knowledge on the past biological responses to temperature alterations and predicting future consequences of these changes on organisms is an active field of research. This may help in the planning of conservation and management actions considering projected global warming^7^.

One of the elements of the global biological system under the influence of temperature changes are pollinators, which provide crucial services for ecosystems’ functioning^8^. Western honey bee *Apis mellifera* (Linnaeus, 1758) is the most widely distributed pollinator species^9,10^, especially valued for its services to agricultural crops^11^. The apicultural industry is also a significant part of the world's economy with a total number of beekeepers only in Europe estimated at 620,000^12^.

It was suggested that global climatic changes can affect honey bee colony fate on multiple levels^13^, including the pressure from its antagonist, the ectoparasite mite *Varroa destructor* Anderson and Trueman^14,15^. *V. destructor* is considered one of the main drivers of *A. mellifera* colony losses worldwide^16^. This mite species parasitizes both imaginal and preimaginal forms of honey bees. It feeds on its host tissues and it needs to enter the bee brood cell to reproduce^17^. Feeding on the bee brood triggers the mite’s oviposition. Newly emerged mites feed on bee pupa, and mated female daughters leave the cell along with their host after it completes its metamorphosis. During this dispersal phase, female mites parasitize adult bees. The hosts are used to disseminate within the bee colony and between colonies^18^. Along with other detrimental effects, *V. destructor* parasitism increases bee virus prevalence and other pathogen transmissions^14^.

The previous study^19^ associated higher winter colony losses with warmer and drier annual conditions. However, the models applied did not include indirect effects (i.e., mite infestation) that could affect winter mortality. The autumn *V. destructor* infestation is one of the main factors affecting survival probability^20–22^ and spring condition^23,24^ of an overwintering honey bee colony. Environmental factors were shown to influence the autumn mite infestation rate stronger than beekeeping management practices^25^. Here we investigate the effect of air temperature variability on the autumn *V. destructor* abundance, controlling the influence of several honey bee colony traits and beekeeping practices. Many studies look for immediate or direct effects of a factor, i.e., at the moment of or just before the period when the response variable is measured. Therefore, the effect of temperature on biological processes is often investigated for an a priori designated time window^26^. For example, in a previous investigation of *V. destructor* population dynamics, a two-month time lag was assumed (e.g. Ref.^27^).

In our approach, we searched for critical moments during the seasons for the temperature factor. We hypothesized that temperature may affect autumn mite populations with different time-lags and directionality. Further, the length of the brood-rearing period, modified by the temperature, can influence the bee abundance and brood availability for mites. Thus, we hypothesized that *V. destructor* abundance can be increased due to the elongation of the brood-rearing period. Alternatively, the extension of the brood-rearing period has no effect on mites’ abundance, but temperature induces other environmental effects (e.g. food availability for bees), independent of the brood-rearing period extent, modulating mite reproduction intensity. To test our hypotheses, we used long-term data series (1991–2020) on the autumn mite abundance in the honey bee colonies. Our results are of importance for our understanding of the drivers of the *V. destructor* population dynamics. They may be used for further prediction of the impacts of global climate change on honey bees and support the management plans associated with the *V. destructor* infestation.

## Materials and methods

### Biological data

We carried out observations of the apiary in the years 1991–1994, 1996–1998 and 2006–2020. The colonies were headed by the queens derived from Carniolan, Caucasian and Buckfast honey bees. The Carniolan and Caucasian queens originated from Polish breeding apiaries and Buckfast queens from Denmark. For simplicity, hereinafter, queen derivation is referred to as a type of bee.

The apiary was located in two places separated by about 40 km: 52.23° N 16.39° E in the years 1991–1999 and 52.41° N 16.33° E in 2006–2020. The two locations were similar in terms of landscape and forage plants. Part of the colonies were transported to bee pasture with buckwheat, lime and/or heather during the season. The number of colonies in the apiary, usually over a dozen, varied occasionally from 3–5 to 17 (Supplementary Table S1).

We carried out treatment of bees in autumn (September–November). To eradicate the *V. destructor* mites from the colonies, synthetic miticides were used, mainly amitraz in the form of fumigation tablets. Occasionally, other synthetic miticides were used. We carried out 3–5 treatments at one-week intervals (i.e. treatment period lasted from 3 to 5 weeks) until no mite-fall could be observed. All colonies in the given year were treated in the same manner. The bees were kept in multi-story hives with a screened bottom board which enabled *V. destructor* mite counts^28^. We collected and counted dead mites the next day after application of acaricide vapour and 1 week after the treatment. We calculated *V. destructor* abundance for each colony as a sum of counted mites from the whole autumn series of treatments. This method provides reliable estimates of *V. destructor* population size and infestation rate in bee colonies^28,29^.

We recorded information about spring swarming, number of colonies merged during summer management of colonies, type of bees and age of queen in each colony. Prior to the autumn *V. destructor* treatment, we counted the number of frames occupied by bees and the frames with capped brood as a measure of bee population size^24^. Moreover, we recorded dates of the first spring cleansing flights and the last autumn flights through direct observations.

### Climatic data

We obtained air temperature data from E-OBS v 20.0 dataset^30^. This dataset presents a European land-only daily high-resolution (0.25° cell) gridded data set for mean surface temperature for the period from 1950 to the present. We aggregated the daily mean values of air temperature over the area 16.25–17.00° E; 52.25–52.75° N covering Site 1 and Site 2 and calculated mean monthly values. Because, during the analysis, the temperature data were available until the 30th of September 2020, three values for October, November and December 2020 were completed using the average of the respective monthly means from the period 2000–2019.

### Data analysis

We used linear mixed-effects models fitted with the Gaussian distribution. We treated information on the *V. destructor* abundance in the colony as a response variable. As potential explanatory variables, we included different biological information regarding investigated colonies and local climatic conditions. Table 1 lists all variables used, specifying the rationale for the incorporation in the modelling. After preliminary analysis, the location effect was not included in the model to avoid wrongly assigning a portion of the variance related to the temperature trend to the location effect. We treated most of the explanatory variables as fixed effects. We included the year as a random term to take account of the non-independent nature of multiple observations (from different colonies within the year) and we estimated synchronized systematic across-colony deviances from the mean mite abundance. We assumed independent and identically distributed errors for year random effects. To correct for heteroscedasticity and non-normality we square root transformed mite abundance prior to the modelling.

**Table 1:** List of variables used in the modelling of the autumn *V. destructor* abundance in the *A. mellifera* colony. The type of the variable, detailed description and proposed hypotheses are specified.

Variable (abbreviation)	Description	Hypothesis
*V. destructor* abundance in autumn (VA)	Response, continuous (No. individuals)	–
Bee abundance in autumn (BA)	Fixed, continuous (No. frames)	Mite abundance increases with adult bee abundance^27^ and strong colonies produce more brood needed for mite reproduction than small colonies^36^
Capped brood in autumn (CB)	Fixed, continuous (No. frames)	Mite abundance increases with bee brood availability^37^ associated with bee abundance^18,23,27^
Number of colonies merged (NC)	Fixed, continuous (No. colonies)	Mite abundance can be influenced, through “reinfestation”^18^, by colonies merging during autumn management practices
Spring swarming (SS)	Fixed, factor (yes:1 or no:0)	Colony fission does not lower *V. destructor* infestation rate in mother colony since division of mite population during swarming is asymmetric^38,39^. Alternatively, swarming creates a broodless period that suspends mite reproduction, and after swarming, colonies show reduced population and brood levels^40^
Type of bees (TB)	Fixed, factor (3 levels: derived from Carniolan, Caucasian or Buckfast honey bees)	Mite abundance can be dependent on the type-specific factors acting directly, e.g. the attractiveness of the brood, grooming or hygienic behaviour^41,42^ and indirectly, e.g. size of winter cluster, onset of brood rearing after winter^43,44^
Age of queen (AQ)	Fixed, factor (3 levels: 1, 2, 3)	Mite abundance can be indirectly affected by the reproductive potential of the honey bee colony depending negatively upon the age of the queen^45,46^, but see:^47^)
Year (YE)	Random, factor (22 levels)	Mite abundance can show interannual variation due to the external environmental drivers and repeated measurements in a year can be internally correlated
Temperature (TE)	Fixed, continuous (°C)	Mite abundance increases with a decrease of temperature—within a certain range—during reproduction of mites^27,48^, but it is hypothesized that temperature can affect autumn mite population with different time-lags and directionality of the relationships, which were subject of the sliding window analysis^26,31,49^
First spring cleansing fligts (SP)	Fixed, continuous (Julian days)	Earlier spring cleansing flights extend the period of activity of bees, therefore can extend the brood-rearing period and availability of brood^15^. Mite abundance increases with bee brood availability^37^
Last autumn flights (AU)	Fixed, continuous (Julian days)	Later last autumn flights extend the period of activity of bees, therefore can extend the brood-rearing period and availability of brood^15^. Mite abundance increases with bee brood availability^37^
Number of active days of bees (DA)	Fixed, continuous (number of days)	Period of activity of bees extends the brood-rearing period and availability of brood^15^. Mite abundance increases with bee brood availability^37^

To test the hypothesis that temperature can affect mite population with different time-lags and directionality of the relationships, we applied a systematic approach of sliding window analysis^26,31^ and identified the optimal time window of air temperature in the prediction of mite abundance. We compared models without air temperature effect with models that additionally incorporate the mean value of temperature from different months (various start dates and durations). We evaluated the strength of temperature signals using the Akaike Information Criterion corrected for small sample sizes (AIC_c_). We used absolute time windows within the range of 15 months (counted back from the end of the year: the 31st of December) to aggregate temperature information. We assumed the linear relationship between response and environmental variables. Two iterations of the sliding window analysis were conducted. In the second iteration the models were refitted including the first (optimal) temperature signal. This approach allowed for the identification of a secondary, suboptimal time window of temperature.

The risk of false positives occurs because of the exploratory nature of the sliding window analysis and a high number of windows tested during analysis^26,31^. Therefore, we conducted 500 randomization tests by reshuffling the date variable in the original response data frame and removing any relationship between temperature and the biological response in order to evaluate the probability of false positives. Such an approach helps to preserve autocorrelation within the climatic data. We compared the distribution of the AIC_c_ values obtained for the best model selected in each random iteration (with no environmental signal) with AIC_c_ of the final model to calculate the probability of observed results being the product of chance.

Pearson's product-moment correlation was used to assess relationships between two identified temperature signals and phenology of the first spring cleansing flights and last autumn flights of bees. Additionally, we tested the potential direct effects of the phenology of the first spring cleansing flights, last autumn flights, and the number of active days of bees on *V. destructor* abundance. We did it by the parallel inclusion of these variables in the developed baseline and final model. We also assessed spatial correlations between random effects of the year (best linear unbiased predictors extracted from the model) and identified temperature signals using European gridded data sets of temperature.

The structure of the fixed effects of the final model was optimized based on the AIC_c_ comparisons. We assessed and satisfied all assumptions of the final model incorporating all explanatory variables (distribution of residuals and overdispersion) with the simulation-based approach using DHARMa package^32^ and checked the correlation between explanatory variables using the variance inflation factor (VIF < 2). We calculated the statistical significance (p), degrees of freedom, and F values of fixed effects using the Satterthwaite approximation. We used lme4^33^, lmerTest^34^ and climwin^26,31^ packages of R^35^.

## Results

**Figure 1:**
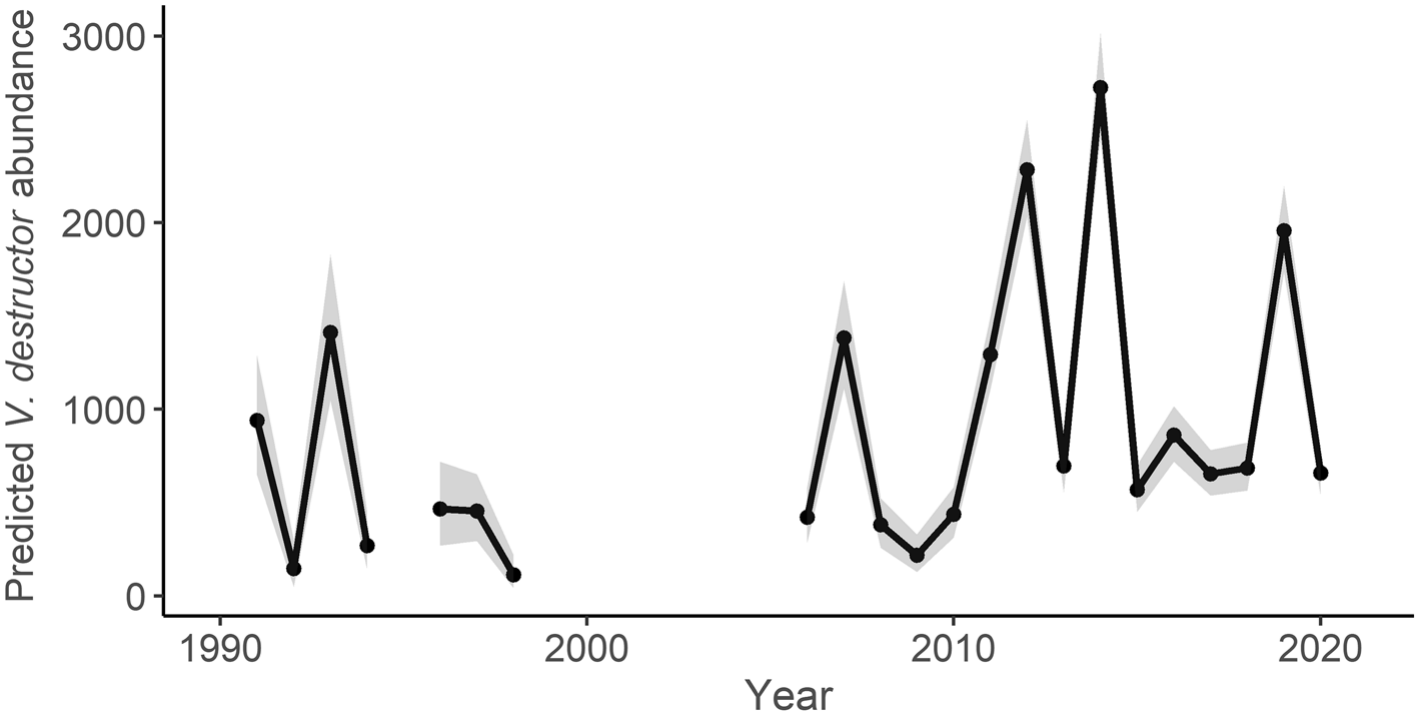
Predicted interannual variation in the *V. destructor* abundance. Estimates of the baseline model for the random effect of the year were used. Shaded areas indicate standard errors of the estimates.

**Figure 2:**
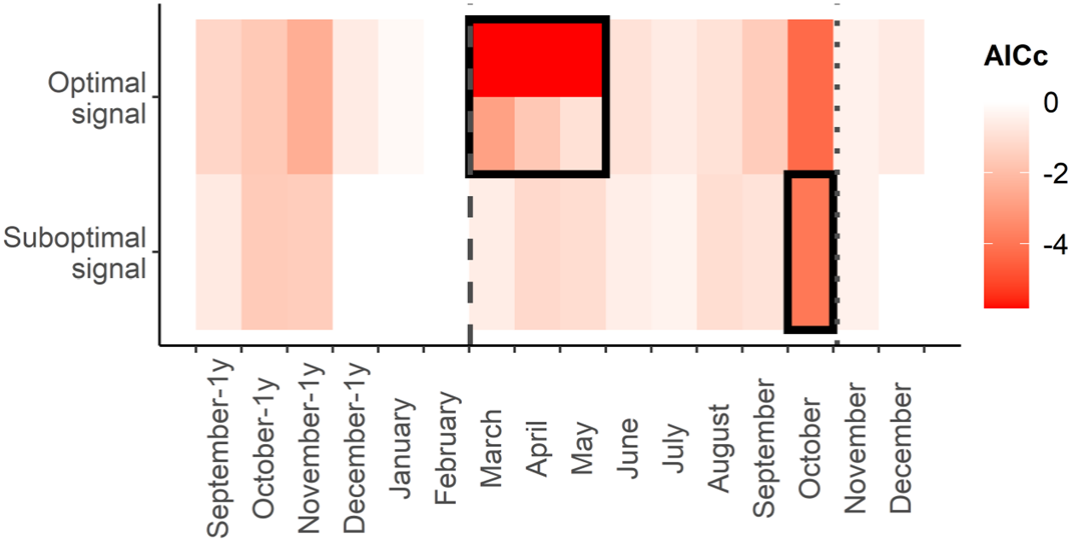
Results of the sliding window analysis indicating optimal and suboptimal signals of the temperature for the prediction of the autumn *V. destructor* abundance. Colour gradient indicate AIC_c_ changes in relation to the baseline model (lower AIC_c_ indicates a better model), black boxes show identified optimal and suboptimal signals, vertical grey lines indicate the multi-year average date of first spring cleansing flights (dashed) and last autumn flights (dotted). Notice that average March–May temperature obtained lower AIC_c_ than averages from single months (AIC_c_ for March–May temperature is indicated in the upper part of the rectangle that shows optimal signal).

We observed strong interannual fluctuations in the *V. destructor* abundance. The random effect of the year explained a large part of the variance in the model of *V. destructor* abundance with calculated ICC = 0.58 (Table 2, Fig. 1). Systematic analysis indicated an optimal time window for the temperature predictor in the period March–May (Fig. 2). The inclusion of this optimal temperature signal significantly improved the model fit (ΔAIC_c_ = − 5.80). The second most important time window of the temperature was identified in October. The inclusion of the suboptimal (second) signal also significantly improved the model fit (ΔAIC_c_ = − 3.95). Randomization tests indicated a high probability of the type I error (false positives) for both temperature signals (p = 0.526 and p = 0.776 for the optimal and suboptimal signal, respectively).

**Table 2:** Parameter estimates of the baseline, temperature, and final model for *V. destructor* abundance. Estimates are given for all fixed effects with standard errors (*SE*). For the random effects residual variance (*σ*^*2*^), the variance associated with tested effects (*τ*), and intraclass correlation coefficient (ICC) are given. Fixed effects of the final model were selected based on the AIC_c_ comparisons. Abbreviations of predictors are given in Table 1. TE is an optimal and TE2 is a suboptimal temperature signal. The number of observations used to fit the models was N = 206 and number of years N_YEAR_ = 22.

Predictors	Baseline model			Temperature model			Final model		
	Estimates	SE	p	Estimates	SE	p	Estimates	SE	p
Intercept	− 3.39	5.77	0.557	− 73.10	24.12	**0.002**	− 72.69	23.86	**0.002**
BA	4.10	0.90	**< 0.001**	4.21	0.89	**< 0.001**	4.31	0.86	**< 0.001**
CB	1.50	0.68	**0.028**	1.63	0.69	**0.018**	1.51	0.67	**0.025**
NC	2.07	0.83	**0.012**	1.93	0.83	**0.019**	1.67	0.81	**0.038**
SS [1]	0.29	2.05	0.889	0.47	2.05	0.817			
TB [Caucasian]	− 6.96	3.71	0.061	− 6.96	3.70	0.060	− 7.63	3.68	**0.038**
TB [Carniolan]	− 2.43	1.78	0.171	− 2.37	1.77	0.181	− 2.99	1.73	0.084
AQ [2]	2.98	1.93	0.121	2.86	1.92	0.137			
AQ [3]	4.98	5.38	0.355	4.57	5.38	0.395			
TE				4.77	2.22	**0.032**	4.87	2.19	**0.026**
TE2				2.87	1.50	0.055	2.87	1.48	0.052
**Random effects**									
σ^2^	100.71			100.59			100.68		
τ_YEAR_	140.45			101.51			98.80		
ICC	0.58			0.50			0.50		
Marginal R^2^/conditional R^2^	0.131/0.637			0.287/0.645			0.288/0.640

**Figure 3:**
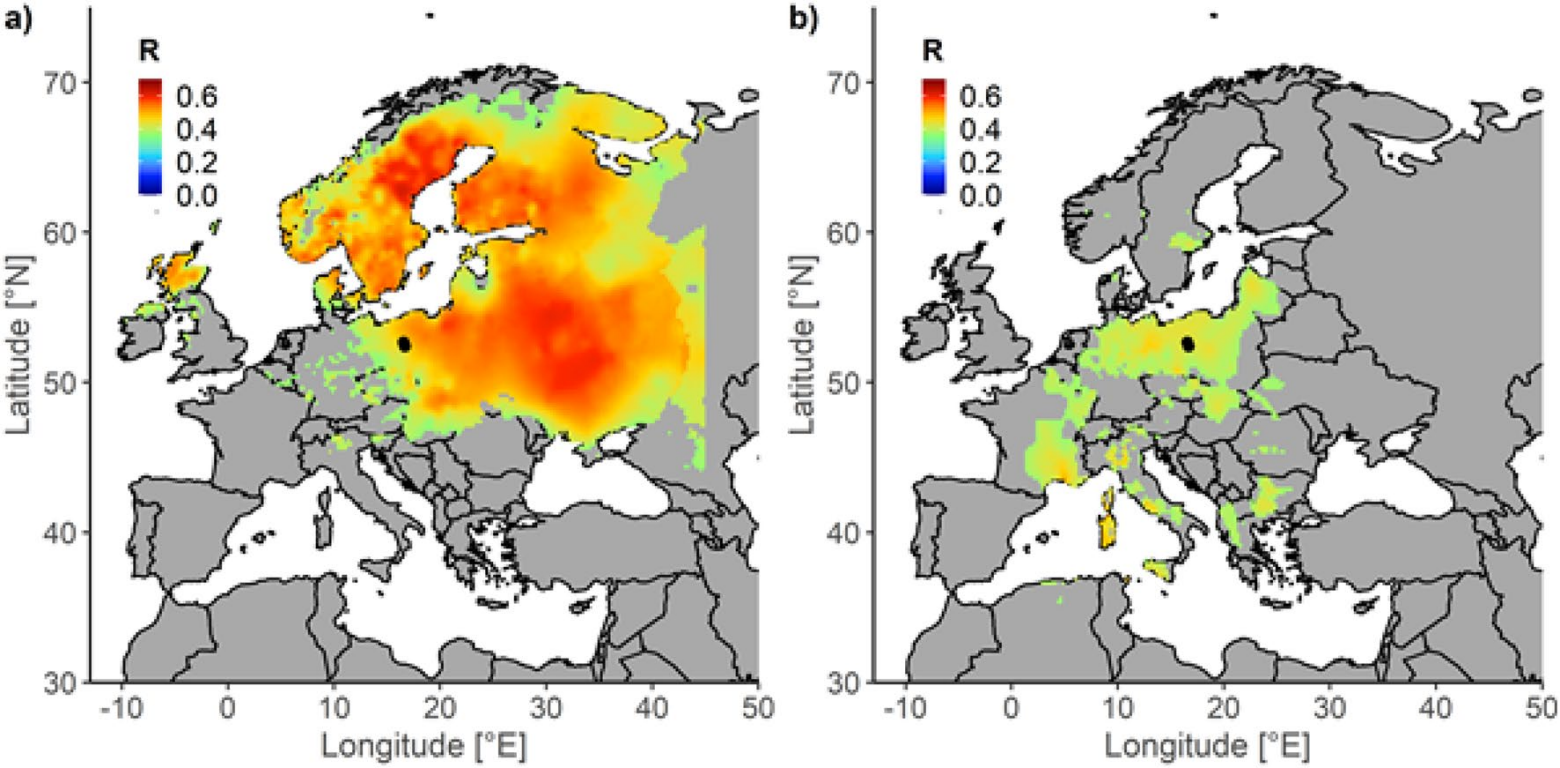
Spatial correlation between estimates of random effects of year and air temperature in the identified critical time windows: March–May (**a**) and October (**b**). Locations of the bee colony are indicated with points. Only cells with significant correlations (p < 0.1) are presented.

We observed a strong spatial correlation (up to r = 0.69, df = 21, p < 0.001) between extracted estimates of random effects of year and temperature in March–May over the large part of central and north-eastern Europe (Fig. 3a). We also observed significant, albeit weaker (up to r = 0.50, df = 21, p < 0.001), correlations between random effects of the year and temperature in October in a more limited region in central Europe (Fig. 3b). These results indicate that the correlation found is not a local, possibly random “patch”.

**Figure 4:**
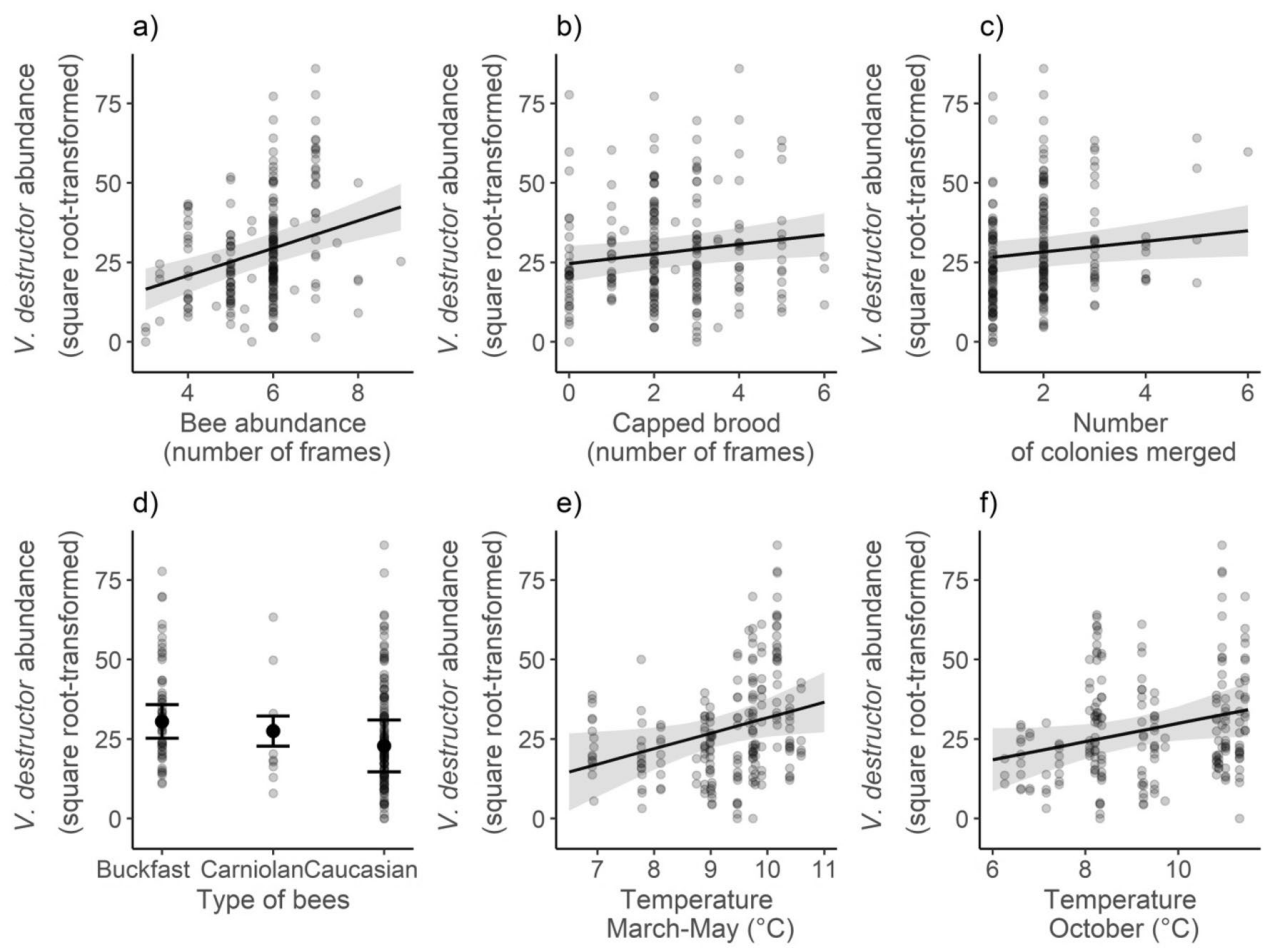
Predicted effect of (**a**) autumn bee abundance; (**b**) autumn capped brood abundance; (**c**) number of colonies merged; (**d**) type of bees; (**e**) identified signal of spring temperature; (**f**) identified signal of autumn temperature on *V. destructor* abundance. Confidence intervals are indicated with shaded areas and bars.

We found significant effects of autumn bee abundance [χ^2^ ( df = 1, N = 206) = 24.84, p < 0.001], autumn capped brood abundance [χ^2^ (df = 1, N = 206) = 5.01, p = 0.025] and number of colonies merged [χ^2^ (df = 1, N = 206) = 4.28, p = 0.038] on autumn *V. destructor* abundance (Table 2, Fig. 4). Significantly lower *V. destructor* abundance was observed in Caucasian bees when compared to Buckfast bees [Estimate = − 7.63, SE = 3.68, t(df = 1183.5) = 2.08, p = 0.039], but there were no significant differences in *V. destructor* abundance between Carniolan and Buckfast [Estimate = − 2.99, SE = 1.73, t(df = 1187.7) = 1.73, p = 0.086] or Caucasian and Carniolan [Estimate = − 4.64, SE = 3.58, t(df = 1184.2) = − 1.29, p = 0.196] bees. All these effects were retained after the final model selection based on AIC_c_. Among the investigated traits of colonies, age of queen [χ^2^ (df = 2, N = 206) = 3.01, p = 0.222], and spring swarming [χ^2^ (df = 1, N = 206) = 0.02, p = 0.889] had no significant effect on *V. destructor* abundance. The final model explained a large part of the variation in the *V. destructor* abundance (64.0%). Incorporation of temperature predictors considerably increased the level of variance explained by the fixed effects from 13.1% (baseline model) to 28.8% (final model). There was a relationship between optimal temperature signal [χ^2^ (df = 1, N = 206) = 4.94, p = 0.026] or suboptimal temperature signal (χ^2^ (df = 1, N = 206) = 3.78, p = 0.052] and *V. destructor* abundance.

**Figure 5:**
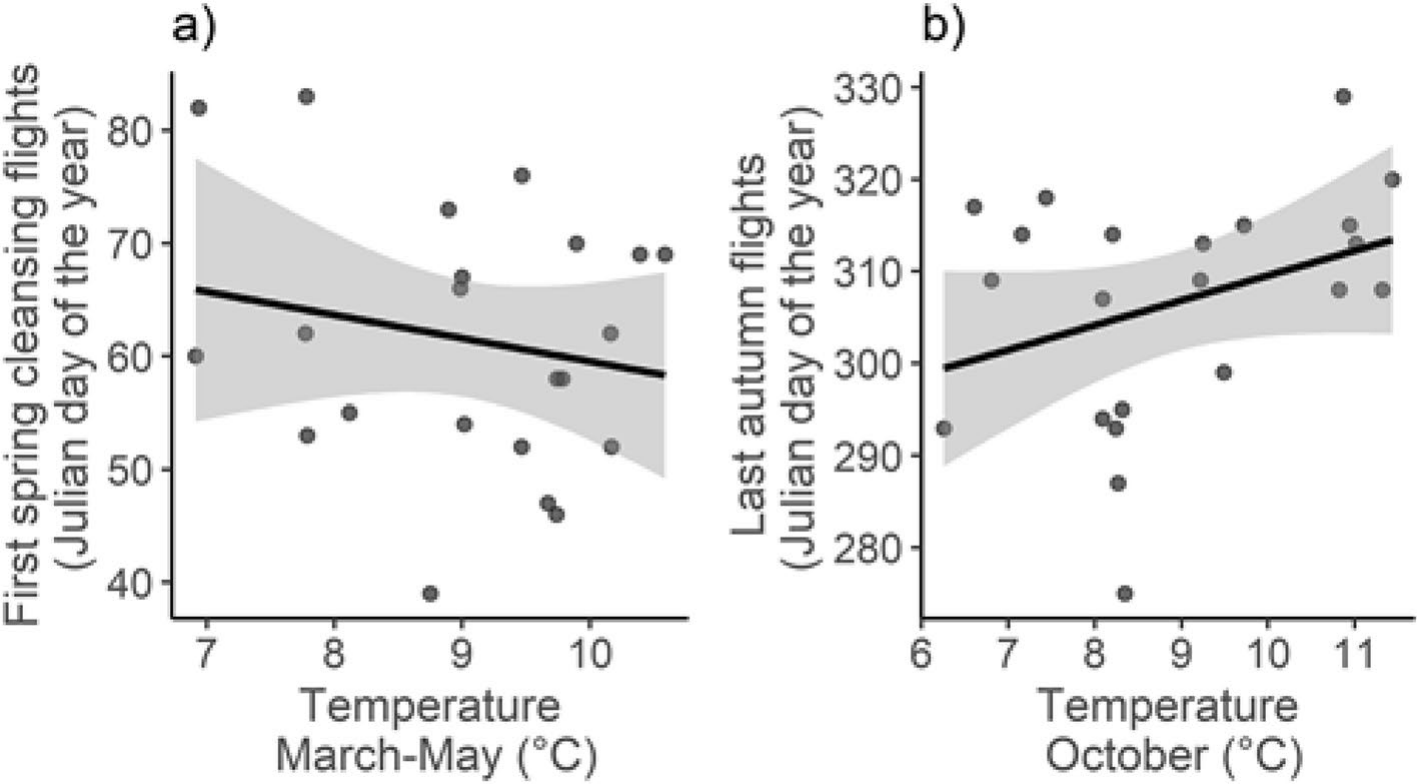
Phenology of the first spring cleansing flights (**a**) and last autumn flights (**b**) of the bees in relation to the temperature conditions during the first (**a**) and the second (**b**) critical time windows.

Correlation between the identified spring temperature signal (March–May) and date of the first cleansing flight was r = − 0.19 (t = − 0.88, df = 20, p = 0.39, Fig. 5a). Correlation between the autumn temperature signal (October) and date of the last flights was r = 0.34 (t = 1.64, df = 20, p = 0.12, Fig. 5b). We found no direct effects of the phenology of the first spring cleansing flights [χ^2^ (df = 1, N = 206) = 0.06, p = 0.806], last autumn flights [χ^2^ (df = 1, N = 206) = 0.60, p = 0.439], or the number of active days of bees [χ^2^ (df = 1, N = 206) = 0.15, p = 0.701] on *V. destructor* abundance. None of these variables did improved the model fit (measured by AIC_c_) after inclusion in the developed baseline or final model.

## Discussion

High *V. destructor* loads preceding summer collapse^22,50^ or wintering^21–23^ were identified as the main risk factors for honey bee colony losses throughout the season. But it is the high infestation rates in autumn that cause more common winter colony deaths and are of serious concern in bee management. *V. destructor* abundance in the autumn is driven by the population dynamics earlier in the season, which, in turn, may depend on temperature conditions^14,27^. In our study, we investigated how temperature shapes autumn *V. destructor* infestation in honey bee. Our results extend previous findings of Switanek et al.^19^ who indicated higher winter honey bee colony losses after warmer and dryer periods. We showed a potential explanation: raised temperatures reinforce *V. destructor* infestations through influence on the bee abundance and brood availability, which in turn, may drive winter colony losses.

Interannual deviances from the long-term mean mite abundance identified in this study were synchronized across-colonies and were potentially associated with common environmental drivers or horizontal spread of mites^51–53^. Besides the climatic impacts, interannual changes in mite infestation may result from fluctuating dynamics in bee and mite survival^21,23^. We may expect that heavy autumn mite infestation rates reduced the number of bees that survived winter. The absence of bee brood throughout several winter months in the Central European climate prevents *V. destructor* reproduction. Therefore, the death rate of bees and mites is expected to be correlated. Regrowth of mite populations follows the rehabilitation of bee colonies. However, the autumn numbers of mites would be smaller than in the previous year, as suggested by Ref.^23^, unless the weak colonies were merged.

In temperate climates, low winter temperatures cause queens to suspend egg laying and a break in brood rearing can be observed^54^. Rise of ambient temperature in late winter triggers the brood onset^55^. Due to the mite reproduction dependence on bee brood presence, intensive brood rearing by the honey bee colony favours the parasite population build-up directly, and indirectly by increasing the adult bee abundance. Although the intensity of bee brood rearing slows down towards the end of the season, the late summer and autumn period may still be important in terms of the availability of bees, particularly the brood, for *V. destructor* reproduction. Indeed, our results showed clearly that the abundance of mites is positively correlated with the abundance of capped brood and bees in the autumn.

Timing of colony activity set off by rise in temperature in late winter should affect timing of swarming^56^. Early rearing of drones, which are produced ahead of swarming, promotes *V. destructor* reproductive success. *V. destructor* prefers reproducing on drone brood than in worker brood due to the longer capping period of the former^57,58^. Therefore, an extended season of colony activity promotes its parasite growth and can eventually handicap the net growth rate of the colony during the season^15^. Similarly, higher autumn temperatures should extend the brood production later in the season, allowing the mites to continue reproduction. We used the first cleansing flight occurrence as a proxy for the onset of brood production^59,60^, but we found no direct relationship between the phenology of the first spring cleansing flights and *V. destructor* abundance in autumn. Surprisingly, we found also no significant effects of the phenology of last autumn flights and the number of active days of bees on measured *V. destructor* abundance. The nonsignificant effect of the bee activity period may result from the potential occurrence of time unfavourable for mite reproduction. For instance, prolonged periods of high summer temperatures may make it difficult to keep the internal temperature of the bee's nest sufficiently low. Therefore, the temperature in some parts of the nest might exceed the mite optimum and hamper its growth^48,61–63^. Our results showed a negative, however not statistically significant, effect of summer temperature. The time windows identified as critical in our analyzes embrace periods of bee activity, i.e. just after the first cleansing flights and just before the last observed bee flights. Temperatures from these periods, though, were not the best predictors for those phenological signs. For example, timing of the first cleansing flight is correlated with the late winter temperatures^59,60^, but also previous summer/autumn temperatures^59^. There is still, however, some trend observed here suggesting indirect relationships between temperature and mite abundance through the phenological changes. On these fringe dates of bee activity, the temperature is low enough to restrict the brood availability for *V. destructor*. However, March–May and October temperatures are high enough to promote brood rearing, and on the other hand, are still lower than those in the middle of the bee activity season. These particular periods, therefore, could assist the growth of the *V. destructor* population because of the rapid increase of parasite hosts, i.e., bee brood and adult bees. *V. destructor* originates from Southeast Asia^64^, which is characterized by warmer climate than Central Europe^65^. Laboratory experimental studies show that species prefer temperatures between 26 and 33 °C, below typical in-brood temperatures of 34.5–35 °C (reviewed in Ref.^18^). Thus, in these colder periods of the year (March–May and October), when air temperatures are ~ 9 °C, positive temperature effects on mite abundance can be expected.

The randomization test indicated a high probability of type I error, where the null hypothesis (no effect of temperature) can be erroneously rejected. While the results of our analysis showed significant effects of temperature in specific periods, similar model support can be obtained by chance due to the high number of time windows tested in the sliding window procedure. However, the importance of temperature signals was revealed both by the primary runs of the sliding window analysis and the further predictors' selection procedure. Importantly, the identified best time windows of temperature coincide with critical periods in the life histories of bees and seem to be biologically reliable. Moreover, a strong spatial correlation was found over the larger area, excluding the probability of random spatial patterns in the temperature data causing spurious correlations. These results provided multiple lines of evidence suggesting the existence of temperature-driven interannual changes in the *V. destructor* abundance. But since our data set is limited to 22 years of observations over three decades and the probability of type I error cannot be excluded, caution is required during the interpretation of the results. Although it is a relatively long period of detailed investigation of bee colonies, even longer time series are needed for a more robust inference on the influences of temperature on *V. destructor* abundance. As the number of colonies is limited over several years of our study, collecting more observations would allow for a more balanced dataset and increase the statistical power of the tests performed.

The temperature effects were the main scope of this study, but further investigation of other climatic components, which can verisimilarly shape *V. destructor* population dynamics and fate of bee colonies, is also advisable. For example, precipitation and winds affect air humidity, and low or high humidity levels experienced during the season may hinder mite reproduction^48^. The change in climate components could also negatively affect bee forage plants and create a pollen shortage. Although honey bees employ several mechanisms, like brood cannibalism, to reduce colony protein demand^66^, pollen shortage has been shown to cause an increase of mite loads^67^. Reduced nectar flow may surcease honey bee brood rearing, but on the other hand, it can also trigger robbing behaviour in bees^68^, fostering, therefore, horizontal transmission of the mites^52^. Moreover, other factors could influence the expansion of the *V. destructor* population, such as drone brood abundance^58,69^ or queen status of the colony^45–47^. However, the beekeeping treatments applied in the investigated apiary allowed us to minimize these effects. For example, there was almost no break in brood production during requeening, because only laying queens were introduced. Drone brood was partially removed from the colonies, while pollen and sugar stores were supplemented in colonies with food shortages. Other possible factors, like colony merging, swarming or queen age, were controlled in the model. Thus, applied beekeeping treatments and modelling approaches allowed us to control most of these confounding effects and identify climatic effects on *V. destructor* abundance. In this study, however, we did not control for the infection through horizontal transmission, as it would require a dedicated study with an isolated apiary and adequate spacing between hives (e.g. Ref.^53^). Horizontal transmission is considered one of the important mechanisms influencing *V. destructor* population dynamics^52^. This mechanism was manifested in our results e.g. as synchronized across-colonies fluctuations. This homogenized response to the common environmental factor of all colonies facilitated the identification of temperature effects.

*A. m. caucasica* subspecies is characterized by slow spring development^43^ in contrast to Buckfast bees that increase numbers rapidly in spring^44^. While it may not be true for every population of these races of bees, this may explain the differences in abundance of *V. destructor* we found between Caucasian and Buckfast derived bees. We found no significant effects of queen age on autumn *V. destructor* abundance. Frequent replacement of a queen, thus managing young queens only, promotes intensive egg laying and in general fosters bee colony health^45,46,70^. Thus, one may expect that mite abundance can be indirectly affected by the reproductive potential of the honey bee colony depending negatively upon the age of the queen. However, previous studies showed that replacement of the queen reduces *V. destructor* infestation risk^47,71^, despite the growth of the mite being tied up with brood availability^18,23,27^.

We hypothesized that swarming should promote bee health with regard to *V. destructor* infestation^40^. Previous studies showed that colonies of mite-resistant *A. mellifera* populations are small and often swarm^72^ and references therein. Swarming is a resultant of the colony strength and nest volume—if the space is too confined, the colony divides. During fission a colony “loses” a considerable number of adults, and a number of mites leave the colony with their hosts. Moreover, a queen leaves with the swarm and consequently there is a broodless period in the mother colony, hampering *V. destructor* population growth, which depends on honey bee brood availability. Therefore, beekeeping practice to prevent bees from swarming, should lead to higher parasite load late in the season. However, in our research we found no significant difference in the infestation levels in the fall between swarming and non-swarming colonies. This is in accordance with the observations of a Gotland mite-resistant population^38^, but also a similar pattern is found in colonies under standard beekeeping management^39^. We found no significant interaction between merging colonies and swarming (Supplementary Table S2). However, the number of colonies merged was an important factor for autumn mite infestation level. In the apiary under investigation, particular care was taken to prevent bees swarming. Swarming episodes were fairly rare, and there was no more than one episode of swarming per colony in the year. On the other hand, the potential effect of a colony and its mite fission can be reduced by (a) merging the colonies that were weakened by division and by (b) drifting and robbing bees^53^.

Keeping colonies strong is one of the beekeeping rules^73^. Numerous colonies allow for high honey yields^74^ and better overwintering^75^. Merging of weak colonies is one of the handling procedures to achieve strong colonies before winter. In our research, some colonies were merged before autumn anti-mite treatments and mite counts. Those colonies, however, experienced higher autumn mite loads. This effect confirms that early mite control, before merging, is advisable. Our results clearly show the importance of beekeeping management practices for *V. destructor* infestation levels. Sustainable management should aim at the balance between keeping strong colonies but preventing high *V. destructor* loads. Predictive models able to integrate honey bee–*Varroa* interactions can be an important tool in the management practices^76^.

Our analyses indicated that mite control can become even more challenging under global climate change. Our results suggested that climatic effects, in addition to management practices, may be one of the main drivers regulating *V. destructor* abundance. We showed that raised spring (March–May) and autumn (October) temperatures reinforce autumn *V. destructor* infestation in honey bee colonies. These effects were potentially associated with the increased bee reproduction in the specific periods of the year and not, as previously hypothesized, with the extended period of activity or accelerated spring onset. Therefore, although the projected further increase in atmospheric temperatures can promote bee brood development, at the same time it can induce a growing risk of honey bee colonies infestation by *V. destructo*r and bee mortality incidental to the mite. Our findings advance the understanding of the complex atmosphere–biosphere interactions and have importance for the studies of other organisms where the temperature may affect population dynamics.

## Supplementary Information

Supplementary Information.

## Data Availability

The datasets generated during the current study are available from the corresponding author on reasonable request.
