# Supplementary material for: Raised seasonal temperatures reinforce autumn Varroa destructor infestation in honey bee colonies
Source: Sci Rep. 2021 Nov 15;11:22256. doi: 10.1038/s41598-021-01369-1 (PMC8593171; doi:10.1038/s41598-021-01369-1)
Supplement: Supplementary file 1 — Supplementary Information. [file 41598_2021_1369_MOESM1_ESM.docx]

**Supplementary materials**

**Raised seasonal temperatures reinforce autumn *Varroa destructor* infestation in honey bee colonies**

Szymon Smoliński^1^, Aleksandra Langowska^2^, Adam Glazaczow^3^

^1^Department of Fisheries Resources, National Marine Fisheries Research Institute, Kołłątaja 1, 81-332 Gdynia, Poland

^2^Department of Zoology, Section of Apidology, Poznań University of Life Sciences, Wojska Polskiego 71c, 60-628 Poznan, Poland

^3^Department of Systematic Zoology, Adam Mickiewicz University, Uniwersytetu Poznańskiego 6, 61-614, Poznan, Poland

Correspondence: Aleksandra Langowska, alango@up.poznan.pl

Table S1. The number of hives observed each year.

| Year | N hives |
| --- | --- |
| 1991 | 3 |
| 1992 | 3 |
| 1993 | 3 |
| 1994 | 4 |
| 1996 | 3 |
| 1997 | 5 |
| 1998 | 5 |
| 2006 | 7 |
| 2007 | 6 |
| 2008 | 8 |
| 2009 | 8 |
| 2010 | 9 |
| 2011 | 13 |
| 2012 | 13 |
| 2013 | 12 |
| 2014 | 13 |
| 2015 | 14 |
| 2016 | 15 |
| 2017 | 17 |
| 2018 | 16 |
| 2019 | 14 |
| 2020 | 15 |

Table S2. Parameter estimates of the final model for *V. destructor* abundance expanded with SS effect and SS*NC interaction. Estimates are given for all fixed effects with standard errors (*SE*). For the random effects residual variance (*σ^2^*), the variance associated with tested effects (*τ*), and intraclass correlation coefficient (ICC) are given. Abbreviations of predictors are given in Table 1 in the main text. TE is an optimal and TE2 is a suboptimal temperature signal. The number of observations used to fit the models was N=206 and number of years N_YEAR_=22.

| Predictors | Estimates | SE | p |
| --- | --- | --- | --- |
| (Intercept) | -72.73 | 23.94 | **0.002** |
| BA | 4.34 | 0.89 | **<0.001** |
| CB | 1.54 | 0.68 | **0.024** |
| NC | 1.50 | 0.89 | 0.090 |
| TB [Caucasian] | -7.48 | 3.72 | **0.044** |
| TB [Carniolan] | -2.88 | 1.76 | 0.100 |
| TE | 4.86 | 2.20 | **0.027** |
| TE2 | 2.89 | 1.48 | 0.052 |
| SS [1] | -2.08 | 4.92 | 0.673 |
| NC * SS [1] | 1.02 | 2.14 | 0.634 |
| Random Effects | | | |
| σ^2^ | 101.62 | | |
| τ _YEAR_ | 99.44 | | |
| ICC | 0.49 | | |
| Marginal R^2^ / Conditional R^2^ | 0.286 / 0.639 | | |
